# Photoinduced Rapid Transformation from Au Nanoagglomerates to Drug‐Conjugated Au Nanovesicles

**DOI:** 10.1002/advs.201700563

**Published:** 2017-12-01

**Authors:** Bijay Kumar Poudel, Jong Oh Kim, Jeong Hoon Byeon

**Affiliations:** ^1^ School of Mechanical Engineering Yeungnam University Gyeongsan 38541 Republic of Korea; ^2^ College of Pharmacy Yeungnam University Gyeongsan 38541 Republic of Korea

**Keywords:** chemo‐photothermal activity, flowing drop reaction, gold agglomerates, nanovesicles, photoirradiation

## Abstract

Gold (Au) agglomerates (AGs) are reassembled using Triton X‐100 (T) and doxorubicin (D) dissolved in ethanol under 185 nm photoirradiation to form TAuD nanovesicles (NVs) under ambient gas flow conditions. The positively charged Au particles are then electrostatically conjugated with the anionic chains of TD components via a flowing drop (FD) reaction. Photoirradiation of the droplets in a tubular reactor continues the photophysicochemical reactions, resulting in the reassembly of Au AGs and TD into TAuD NVs. The fabricated NVs are electrostatically collected onto a polished aluminum rod in a single‐pass configuration. The dispersion of NVs is employed for bioassays to confirm uptake by cells and accumulation in tumors. The chemo‐photothermal activity is determined both in vitro and in vivo. Different combinations of components are also used to fabricate NVs using the FD reaction, and these NVs are suitable for gene delivery as well. This newly designed gaseous single‐pass process results in the reassembly of Au AGs for incorporation with TD without the need of batch wet chemical reactions, modifications, separations, or purifications. Thus, this process offers an efficient platform for preparing biofunctional Au nanostructures that requires neither complex physicochemical steps nor special storage techniques.

## 1 Introduction

Plasmonic gold (Au)‐based nanostructures (NSs) are frequently employed for therapeutic and diagnostic applications because they are chemically inert, can be easily prepared, are highly biocompatible, and have tunable optical properties (i.e., localized surface plasmon resonance (LSPR) effect).^1^ In particular, the strong light absorption of NSs in the near‐infrared (NIR) region generates nonradiative energy dissipation through Landau damping, producing sufficient heat for photothermal hyperthermia therapies. NSs have been extensively employed in such therapies using a variety of treatment modes and different bioactive molecules, such as drugs and targeting agents.^2^ Changing the shape of Au NSs has been reported to manipulate the LSPR wavelength from visible to NIR. Compared with the very limited red‐shift in LSPR absorption resulting from increasing Au size (the simplest method), this change results in stronger optical behavior in photothermal therapy and optical diagnosis.^3^ Au nanorods (NRs) are frequently employed to achieve red‐shifting or broadening, and the absorption spectra can be tuned by controlling the aspect ratios of Au.^4^ More recently, Au NRs have been reported to limit cell viability because of physical features, such as sharp edges, tips, and residues (e.g., cetyltrimethylammonium bromide), and have low in vivo retention time caused by high aspect ratios resulting from their anisotropic characteristics.^5^, ^6^ To address these issues, Au nanovesicles (NVs) have been deliberately fabricated to induce strong plasmonic coupling between primary particles of Au agglomerates (AGs), thereby increasing their LSPR absorption for effective NIR‐induced photothermal therapy.^7^, ^8^ Furthermore, the optical properties of other Au NSs, such as nanostars, nanocages, nanoshells, and nanopopcorns, also have been manipulated or tuned to allow for their effective biomedical application.^9^

Because of their essential plasmonic properties and biocompatibility, engineered Au NSs have been increasingly used in a broad range of theranostic applications.^10^ Specifically, engineered Au is commonly prepared from Au nanoparticles (Au NPs) via seed‐mediated or ‐assisted multistep hydrothermal approaches. Further growth of Au on seed NPs can be catalytically or galvanostatically initiated to form the desired Au NSs. Au ions from a dissolved precursor (e.g., chloroauric acid) are reduced during the hydrothermal reaction to yield Au atoms that are attached to the seed surfaces. These atoms are subsequently deposited as Au branches that can be designed by inducing specific or asymmetric facet growth in the presence of reducing agents (e.g., trisodium citrate, sodium borohydride) and surfactants or capping molecules.^11^ To employ these Au NSs in specific biomedical applications, bioactive molecules, such as drugs, targeting agents, protective coatings, or porous supports must be linked as biofunctional Au nanomaterials (NMs) to improve their theranostic effects.^12^ Unfortunately, these NS posttreatments make the syntheses more complex, time‐consuming, and expensive and require many additional reagents, purifications, or separations.^13^, ^14^, ^15^ Moreover, decreasing or rationalizing the unwanted cytotoxicities of these fabricated nanocomposites is a great challenge to their efficacious use in biomedical applications.^16^

Although state‐of‐the‐art nanoformulations are beginning to expand the number of realizable nanomedicines, the efficient manufacturing of on‐demand biofunctional NMs remains a significant limitation. Obstacles hamper the streamlined approval of such technologies for clinical use and for nanomedicinal and pharmaceutical applications.^17^ The difficulty of continuously manufacturing increasingly complex systems is also a significant challenge that continues to prevent the translation of bench‐top discoveries into promising technologies.^18^ Therefore, it is highly desirable to develop continuous, realizable, and built‐to‐order methodologies for manufacturing biofunctional NMs that possess the necessary physicochemical properties for integration into nanomedical systems or treatments.

**Figure 1:**
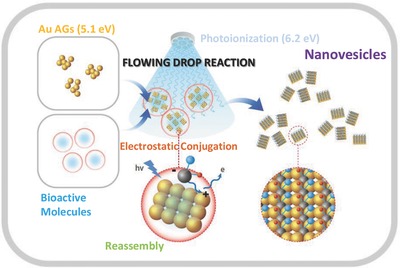
Schematic of the reassembly of Au AGs with bioactive molecules (i.e., TD) in ethanolic droplets under photoirradiation (UV, 185 nm). Photon energy (6.2 eV) higher than the Au work function (5.1 eV) induces Au photoionization (i.e., positively charged via electron loss), and the charged Au electrostatically conjugates with negatively charged groups in TD. The positive charging and subsequent TD conjugation drive the reassembly of Au AGs into NVs (TAuD).

Herein, we developed a novel and efficient approach for continuously manufacturing multifunctional Au NVs for chemo‐photothermal therapy via reassembly of Au AGs (a common form of Au NSs produced for storage) in a gaseous single‐pass configuration. This method requires neither hydrothermal reactions, separations, purifications, or post‐treatments for incorporation with bioactive molecules nor supporting/cargo materials. All required components for the Au‐based chemo‐photothermal therapy are included as a form of NV in the single‐pass gas flow under ambient conditions. Au photoionization in a flowing drop (FD) by irradiation with a photon energy (6.2 eV) higher than the Au work function (5.1 eV) shifted the Au surface into an active state for electrostatic conjugation with bioactive molecules in an FD. The electron loss from the primary Au particles in AGs renders them positively charged and induces rearrangements of the gap spaces in primary particles via repulsive forces between the positively charged surfaces.^19^ Suspended bioactive molecules (containing negatively charged functional groups) near the primary particles were incorporated into the gap spaces^20^ and electrostatically conjugated with the Au surfaces (**Figure** **1**). Briefly, Au AGs were produced via spark ablation between two pure Au rods, and a Triton X‐100 (T) and doxorubicin (D) mixture was chosen as the bioactive molecule to test the feasibility of the FD reaction as an on‐demand manufacturing platform. T has been successfully employed to arrange primary Au particles into multilayered Au nanobunches in a controllable manner.^21^ The positively charged surfaces of the primary Au particles were rearranged via photoionization and conjugated with the phenyl ether groups (as electron donors)^22^ of TD in FDs; thus, the AGs were reassembled, and TD was incorporated into the voids between the primary particles to form TAuD NVs. The fabricated NVs were then tested in both in vitro (MDA‐MB‐231 and MCF‐7 cells) and in vivo (MDA‐MB‐231 bearing BALB/c mice) assays to evaluate their efficacy in chemo‐photothermal therapy (Figure S1, Supporting Information). The conjugation enhanced internalization of NVs into cells. The NIR responsiveness (both 632 and 808 nm wavelengths) of NVs owing to plasmon coupling (broadband light absorption) between the adjacent primary particles facilitated the spatial and temporal controls of D release, resulting in significant reductions in tumor volume without apparent systemic toxicity. The use of lipid molecules instead of T fabricated a different combination of NVs, which were also suitable as nanocarriers for gene delivery.

## 2 Results and Discussion

**Figure 2:**
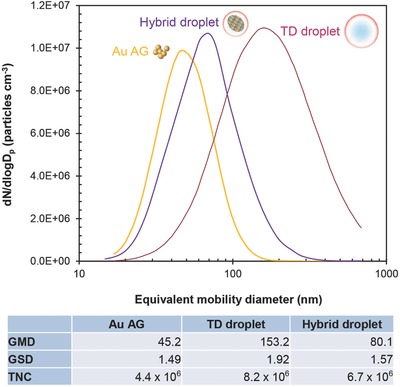
Size distribution of Au AG (from spark ablation of Au rods), TD droplets (TD dissolved in ethanol), and hybrid droplets (Au AG including TD droplets) under 185 nm UV irradiation before solvent extraction via diffusion dryer. Summaries of the distributions are noted at the bottom.

The formation of hybrid droplets from the FD reaction was verified by measuring the size distribution of Au AGs, TD droplets, and their merged forms (hybrid droplets) using a scanning mobility particle sizer (SMPS, 3936, TSI, USA) before solvent extraction in the denuder (Figure S1, Supporting Information). The AGs were formed by Brownian motion (thermal collision behavior) of primary Au particles (formed by the condensation of spark‐ablated Au vapors) in the presence of compressed nitrogen gas flow. The TD droplets were generated by collison atomization of an ethanolic TD solution by injecting another compressed nitrogen gas flow. The hybrid droplets were prepared by direct injection of the Au AG‐laden gas flow into the collison atomization device as the working fluid, where the AGs were incorporated into the TD solution to be atomized as the hybrid droplets. The hybrid droplets were directed to a tubular reactor under 185 nm UV irradiation. The size distributions of the AG, TD, and hybrid droplets, measured using SMPS, are shown in **Figure** **2**. The results are presented as the geometric mean diameter (GMD), geometric standard deviation (GSD), and total number concentration (TNC). The hybrid droplet size also showed a unimodal configuration (located between the distributions of AG and TD) similar to individual AG and TD cases, although there were significant differences between the AG and TD cases. This result implied that nearly all AGs were included in the TD droplets during the atomization process; thus, this product was suitable for use in the subsequent FD reaction. Interestingly, the GSD of hybrid droplets was significantly lower (narrower) than that of the TD droplet, possibly caused by the reassembly of AGs with TD components (intervening and subsequent conjugating) under photoirradiation, including shattering of the AGs during atomization.^23^ This result suggested that the photophysicochemical reassembly of Au AGs is a suitable route for fabricating functionalized Au NSs.

**Figure 3:**
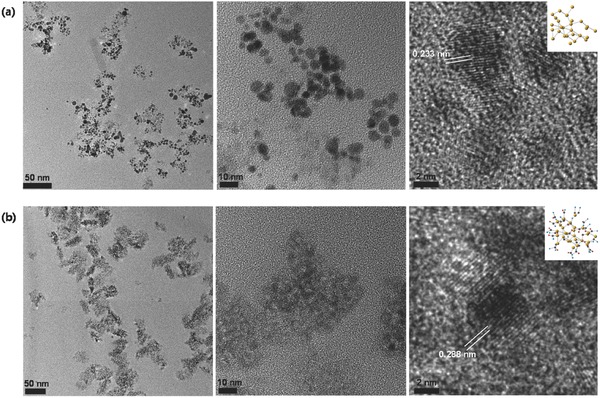
Low‐ and high‐magnification TEM images of a) Au AGs and b) TAuD NVs after passing through a diffusion dryer. The right‐side images of both samples include interplanar distances and plausible microscopic surface structures (yellow, Au; blue, T; and red, D). The Au AGs are seen as clusters of dark dots (primary Au particles, ≈6 nm), whereas TAuD NVs comprise contrasting elements, dark dots (reassembled primary Au particles, ≈4 nm), and brighter layers (conjugated TD). In the case of TAuD, the positions of dots are confined within a brighter layer as the form of NV.

The reassembly was also validated by transmission electron microscope (TEM, CM‐100, FEI/Philips, USA) measurements. As shown in **Figure** **3**a, particles resulting from spark ablation were AGs of primary Au particles (≈6 nm in lateral dimension) caused by Brownian motion during suspension in the gas flow. According to image analysis of 400 AGs, the average size of a single isolated AG was ≈48.8 nm, a result consistent with the size acquired from SMPS measurements. The primary particles were spherical, had smooth surfaces, and had clear voids between the particles, implying that the AGs comprised only Au particles. High‐magnification TEM showed a *d*‐spacing of 0.233 nm for adjacent lattices, corresponding to the Miller (111) face‐centered cubic plane; thus, this result further supported the fact that the AGs were derived from pure primary Au particles. Upon AG photoionization with TD (after solvent extraction in the denuder), the particle morphology significantly changed, appearing as dark dots tightly surrounded by a brighter contrast layer (Figure 3b). The dark and light components correspond to Au particles and TD, respectively, and the tightly packed structure within the different material (i.e., vesicular structure) may result from electrostatic interaction between the positively charged Au surfaces and the negatively charged functional groups of TD during photoirradiation. According to image analysis of 400 NVs, the average lateral dimension of isolated TAuD NVs is 88.2 nm, which is consistent with the SMPS measurement. To measure the hydrodynamic size distribution of the NVs, a dynamic light scattering (DLS, Nano‐ZS, Malvern Instruments, UK) analysis was employed after the collected NVs were dispersed in phosphate‐buffered saline (PBS) (0.01 m, pH 7.4). The average size was found to be 138 nm (Figure S2a, Supporting Information), which is larger than that obtained from the SMPS measurement. This size discrepancy between the two methods might have resulted from the significantly higher NV concentration used in the DLS measurement. Nevertheless, the average hydrodynamic size was ≤200 nm, probably due to the overall positive potential (+7.8 mV). Thus, the fabricated NVs are suitable for enhanced permeability and passive accumulation into tumor tissues via fenestrations and subsequent internalization via nonspecific adsorptive endocytosis.^24^ The analysis of high‐magnification images indicated particles of different sizes (≈4 nm) and *d*‐spacing (0.288 nm), a result consistent with the replacement of Au atoms on Au particle surfaces by other elements of TD during photoirradiation that reassembles the AGs into TAuD NVs. The UV–vis (T60, PG Instruments, UK) spectra of the fabricated TAuD NVs including TAu NVs and Au AGs (Figure S2b, Supporting Information) dispersed in PBS (Figure S2b, inset) showed peaks at 400–550 nm that can be attributed to the TD incorporation and LSPR effect of Au NPs (absorption wavelength, 520–530 nm). Although the Au AGs show only an absorption peak at ≈550 nm, the TAuD or TAu NVs show a broadband absorption spectrum after Au AGs are reacted with TD or T under photoirradiation. This result may be caused by the rearrangement of primary Au particles in hybrid droplets, facilitating interparticle plasmonic coupling of adjacent Au particles.^25^, ^26^ Unlike a selective NIR absorption,^27^ the fabricated NVs were consistent with previously reported Au NVs.^28^, ^29^ Even though the previous reports required incorporation of NIR responsive polymers to derive the broadband absorptions, the current NVs achieved similar absorption spectra even without the polymers. This may encourage combined therapies with appropriate electronics or nanoscale fluorescent additives to facilitate advanced mapping, sensing, and localized photoinduced therapies for efficient cancer treatments.^30^ The stability results of size distribution and zeta potential after the TAuD NVs were dispersed in PBS are shown in Figure S2c in the Supporting Information. There were no significant changes in the size distribution and zeta potential for 15 d measurements. In addition, for fabricating TAu, T concentration of 2.5% v/v (in ethanol) was chosen to avoid unwanted increases in size and cytotoxicity and prevent the formation of loose NV structures into undefined AGs (Figure S3, Supporting Information).

**Figure 4:**
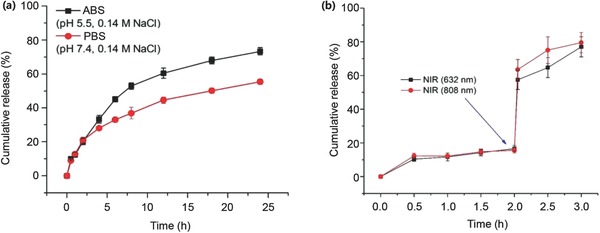
In vitro release profiles of D from TAuD NVs. a) pH‐triggered (5.5 and 7.4) (for 24 h) and b) NIR‐triggered (632 and 808 nm) (for 3 h, irradiated for 5 min after 2 h) D release.

The surface chemistry of the fabricated NVs was analyzed to verify the photoirradiation‐induced conjugation between Au and TD. **Figure** **4**a shows the Au 4f X‐ray photoelectron spectroscopy (XPS, K‐Alpha, Thermo Scientific, USA) results of Au AG and TAuD samples. The AG has two distinct peaks, Au 4f_5/2_ and Au 4f_7/2_ at 89.2 and 85.5 eV (separated by 3.7 eV from the spin–orbit splitting), respectively. These peaks are consistent with those of Au^0^. Analogous data for the TAuD sample were 86.6 and 83.2 eV (separated by 3.4 eV and corresponded to Au^3+^), which have respective negative shifts of 2.6 and 2.3 eV for Au 4f_5/2_ and Au 4f_7/2_ caused by charge transfers between Au and TD, suggesting that photoirradiation results in electrostatic conjugation between Au and TD. In Fourier transform infrared (FTIR, iS‐10, Thermo Electron, USA) analyses (Figure S4b, Supporting Information), the conjugation between Au and TD was further verified by comparing TAu and AuD with TAuD. The TAu spectrum exhibited a characteristic band at 1512 cm^−1^, corresponding to the coupling of aromatic C—C stretching and C—H— deformation modes of T in TAu. This band was also present in the TAuD spectrum but not in the AuD spectrum. The broadband at ≈1610 cm^−1^ in AuD and TAuD could be assigned to the bending of the N—H groups of D, implying that TD was successfully incorporated into Au surfaces by photoinduced electrostatic conjugation. The Raman spectrum of TAuD (Figure S4c, Supporting Information) showed a sharp peak at 1648 cm^−1^ that was not present in the Au AG and TD spectra. This result suggested that the Raman signal of D (corresponding to the hydrogen bond of the C=O group in ring B)^31^ was increased by Au photoionization in TAuD. These surface chemistry analyses provide realizable scientific potentials based on photoionization for restructuring, reassembling, and functionalizing Au particles in gas flow at ambient conditions (i.e., a simple, green, and continuous manufacturing platform).

Because we chose a multifunctional application of the fabricated NVs to cancer tissues as chemo‐photothermal therapy, the drug‐release characteristics of the NVs were evaluated in different buffer solutions (PBS, pH 7.4; acetate‐buffered saline (ABS), pH 5.5) and exposed to different NIR wavelengths (808 and 632 nm). The release of D from the NVs (D loading fraction of 31.6 ± 5.5% w/w in TAuD) was observed to be pH‐dependent, with 50% cumulative release at pH 7.4 (in PBS) and 70% at pH 5.5 (in ABS) over 24 h (Figure 4a). The higher aqueous solubility of D (*pK*_a_ ≈ 8.3) at pH 5.5 was thought to have contributed to its faster release.^32^ This result was highly relevant to the acidic environment of endolysosomal compartments (pH 4.5–5.0), where NVs are internalized by cancer cells. Next, the photothermal elevation in temperature upon NIR irradiation was investigated. Heat generation was found to be both concentration‐ and irradiation intensity‐dependent for irradiation at both wavelengths, 808 and 632 nm (Figures S5 and S6, Supporting Information), without significant differences between wavelengths. This photoinduced heat generation represented drastic increases in D release upon NV exposure to NIR (4 W cm^−2^) for 5 min, with the cumulative D release increasing from 16% to ≈60% (Figure 4b). This result suggested that the fabricated NVs can be employed for phototriggering controlled release applications.

**Figure 5:**
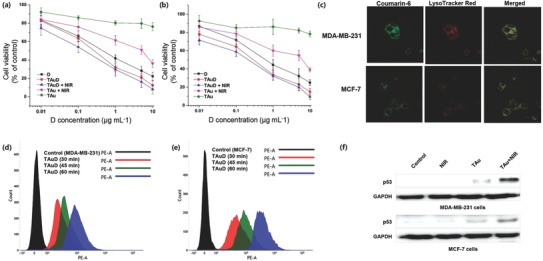
In vitro anticancer effects for TAu and TAuD in the absence and presence of NIR irradiation, including free D, on a) MDA‐MB‐231 and b) MCF‐7 cells. c) CLSM images of coumarin‐6 loaded TAu in MDA‐MB‐231 and MCF cells. FACS analyses showing cellular uptake of TAuD by d) MDA‐MB‐231 and e) MCF‐7 cells. f) Western blot analysis of p53 in the cells treated with TAu in the absence and presence of NIR (808 nm) irradiation.

In vitro anticancer effects were evaluated by cytotoxicity measurements in MDA‐MB‐231 (**Figure** **5**a) and MCF‐7 (Figure 5b) cells after 24 h incubation using 3‐(4,5‐dimethylthiazol‐2‐yl)‐2,5‐diphenyltetrazolium bromide (MTT) assays. The viability of cells treated with TAu was greater than 75% in both cell types, indicating that TAu before D loading is biocompatible. Although T is known as a membrane‐lytic agent, it was present in sublytic concentrations (<0.01% w/w). TD incorporation into Au under photoirradiation and subsequent solvent extraction was confined to thermally stable amorphous T within nanometric spaces.^33^, ^34^ The IC_50_ values for MDA‐MB‐231 and MCF‐7 cells upon TAuD administration were 0.24 and 0.27 µg mL^−1^, respectively. These values were significantly lower than those of free D (MDA‐MB‐231, 0.48 µg mL^−1^; MCF‐7, 0.77 µg mL^−1^), suggesting that TAuD increased cellular uptake and retention of D and bypassed transporter‐mediated efflux of D.^35^ Upon NIR irradiation of TAuD‐treated cells, the IC_50_ values further decreased in both cell types (MDA‐MB‐231, 0.13 µg mL^−1^; MCF‐7, 0.15 µg mL^−1^). This result might have been caused by hyperthermia‐induced burst drug release, chemosensitization, and thermal damage upon irradiation. Cellular uptake of TAu was studied using confocal laser scanning microscopy (CLSM, Leica Microsystems, Germany) (Figure 5c) and fluorescence‐activated cell sorting (FACS, BD Biosciences, USA) (Figure 5d,e). The green fluorescent signal from coumarin 6‐loaded TAu colocalized with red lysosomal dye, indicating lysosomal uptake through endocytosis. The red fluorescence of D was not used because of its relatively weak fluorescence, possibly caused by quenching^34^ due to interaction and/or aggregation between Au primary particles in the presence of T. The intracellular accumulation of D was confirmed by FACS, demonstrating that uptake was time‐dependent. Fractions of TAuD‐treated cells (with and without NIR irradiation) in different apoptotic phases were ascertained by the measurements (Figure S7, Supporting Information). After incubation with TAuD, the proportion of cells in early and late apoptotic phases was significantly larger than that of cells treated with free D. These proportions further increased with NIR irradiation in both cell types. A western blot analysis (Figure 5f) was employed to investigate cell death by necrosis induced by NIR irradiation and the expression of the apoptotic levels of apoptotic marker p53 was measured. TAu‐treated cells expressed higher p53 levels than controls and NIR‐irradiated (TAu‐untreated) and nonirradiated (TAu‐treated) cells, which is consistent with the findings of apoptosis assays. This result indicated that no significant necrosis occurred in TAu‐treated cells under NIR irradiation because cell death by photothermal effects is more immediate than that from conventional chemotherapy or radiotherapy. Thus, the fabricated NVs offer efficient anticancer chemo‐photothermal therapy in an apoptosis‐dominant (rather than coagulative necrosis) configuration that is controlled by modulating the NIR irradiation (intensity and duration) and the dose and composition of photoinducers (e.g., NVs); thus, inflammatory responses could be minimized.^36^, ^37^

**Figure 6:**
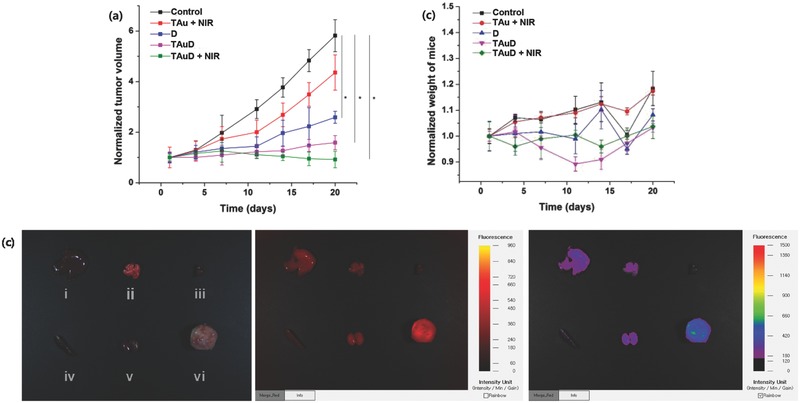
In vivo antitumor evaluations. a) Tumor growth and b) weight‐change profiles in MDA‐MB‐231 bearing xenograft mice for TAu and TAuD in the absence and presence of NIR irradiation, including free D. c) Ex vivo fluorescence images of Cy5.5 alone (middle) and Cy5.5‐conjugated TAu (right) for major organs i) liver, ii) lung, iii) heart, iv) spleen, and v) kidney,) and vi) tumor harvested from euthanized mice 24 h after injection.

The live/dead assay (Figure S8, Supporting Information) was conducted on TAu‐treated MDA‐MB‐231 cells exposed to NIR irradiation (≈2 mm beam diameter, 4 W cm^−2^). The beam‐exposed region exhibited cell death in TAu‐treated cells but not in control (NIR only) or TAu‐treated cells without NIR exposure, suggesting that photothermal cell killing was site‐specific. Cell cycle analysis of MDA‐MB‐231 cells after treatment with free D and TAuD was performed using redox dye (Figure S9, Supporting Information). Most control cells were in S and G0/G1 phase, and free D treatment led to G2/M growth arrest in 53% of cells. Among TAuD‐treated cells, 67% reached the G2/M check‐point. This observation might have resulted from increased TAuD internalization into endolysosomes, followed by D release into nucleus with concomitant reduction in D efflux via cell membrane transporters. The antitumor efficacy of TAu and TAuD with NIR irradiation was investigated in vivo using MDA‐MB‐231‐bearing female BALB/c nude mice (**Figure** **6**a). Compared with untreated controls, mice treated with free D or TAu or TAuD with irradiation exhibited a significant reduction in tumor volume by the end of the experimental period. TAu‐treated, irradiated mice also exhibited significant tumor regression resulting from hyperthermia induction. Weight‐change profiles (Figure 6b) demonstrated that TAuD treatment with irradiation did not induce significant changes in the weights of mice by the end of treatment, whereas free D treatment resulted in weight loss starting at 7 d of treatment. This finding suggested that TAuD treatment did not induce significant systemic cytotoxicity associated with D. TAu biodistribution at 24 h after injection was assessed using ex vivo fluorescence imaging (FOBI, NeoScience, Korea) (Figure 6c). TAu was found to accumulate in the tumor, liver, lung, and kidney, and its accumulation in the heart and spleen was minimal. The mean fluorescence intensity of Cy5.5‐conjugated TAu in tumors was double of that in the liver, suggesting the preferential and passive accumulation of TAu in tumor tissues because of its enhanced permeation and retention afforded by the TAu size distribution. In vivo imaging was performed to investigate the photothermal effect of TAuD treatment in MDA‐MB‐231‐bearing xenograft mice. At 24 h after injection via the tail vein, tumors were exposed to NIR irradiation (4.0 W cm^−2^) for up to 5 min (Figure S10, Supporting Information). No significant thermal elevation occurred in mice treated with saline alone. Mice treated with TAuD exhibited significant thermal elevation in the focal region, from 34.8° to 43.4 °C. No obvious temperature changes were observed in non‐NIR‐exposed body parts. Even though NIR light can only penetrate a few millimeters deep in biological organisms,^38^ the results showed pronounced anticancer effects, probably due to the effective targeting of tumors by TAuD.

**Figure 7:**
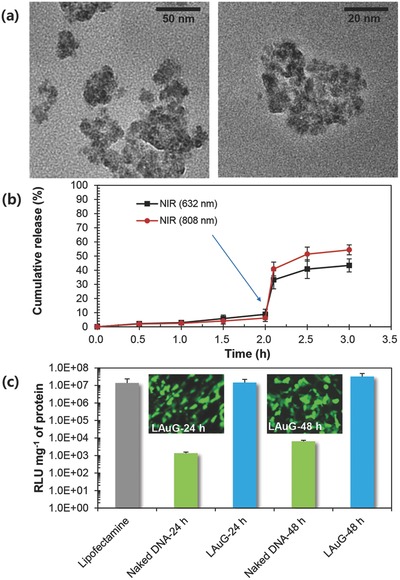
Other combinations to fabricate different types of NVs by employing lipid molecules (i.e., LAu) instead of T. a) Low‐ and high‐magnification TEM images of LAu from FD reaction. b) NIR (632 and 808 nm, for 5 min)‐triggered D release profiles from LAuD to verify photothermal activity of LAu. c) Gene delivery performances of pDNA (EGFP included) complexed LAu (i.e., LAuG) compared with naked DNA and commercial lipofectamine. Insets show fluorescent images of HeLa cells for 24 and 48 h incubation with LAuG.

Other NVs, including lipid (L‐1118, Echelon Biosciences, USA) molecule‐conjugated Au (LAuD) and gene‐complexed LAu (LAuG), were fabricated via the FD reaction. **Figure** **7**a shows the shapes of LAu and reveals that darker spots (Au) are trapped within brighter contrast materials (L), suggesting that the FD reaction can be extended to fabricate different types of NVs without complex system modifications. The NIR‐triggered D release (Figure 7b) was tested for LAuD, and the results are similar to those for TAuD. The gene delivery performance of LAu into HeLa cells was further evaluated using plasmid DNA (pDNA) containing genes for luciferase and enhanced green fluorescent protein (EGFP) as another therapeutic application of the FD reaction (Figure 7c). The amount of LAu–pDNA complex (i.e., LAuG) transfected into cells (relative luminescence unit (RLU) per mg) after 24 and 48 h incubation was comparable to that of lipofectamine and significantly greater than that of naked DNA. Figure 7 insets show the corresponding fluorescent images (derived from EGFP expression) after 24 and 48 h incubation, which further verify the gene delivery performance. Considering the average size of LAu NVs (103.8 nm), the transfection of pDNA was probably due to receptor‐mediated macropinocytosis or transient membrane pores through the cytoplasm of cells in accordance with previous reports.^39^, ^40^ Even though the transfection efficiencies of the NVs were comparable with lipofectamine owing to the protection against direct exposure of pDNA to the intracellular trafficking enzymes, the efficiencies were still smaller than those from cationic polymer‐ or lipid‐based gene carriers^41^, ^42^ because of no polycationic addition compounds (+2.9 mV, zeta potential). By the way, the results of NIR‐triggered D release and gene delivery using LAu further demonstrated the potential of the FD reaction. Thus, NVs with various components may be efficiently fabricated for a broad range of nanomedicinal applications.

## 3 Conclusion

D‐loaded Au NVs were fabricated via the FD reaction in an ambient single‐pass configuration without the use of any hydrothermal chemical processes. The application of photon energy higher than that of the Au work function to hybrid droplets containing Au AGs and bioactive molecules (T and D) caused the reassembly of Au AGs and electrostatic conjugation between positively charged Au surfaces and negatively charged functional groups of bioactive molecules, resulting in the formation of biofunctional NVs. The NVs demonstrated biocompatibility, broadband photoconversion, and pH‐ and NIR‐triggerable D release. Thus, the NVs were suitable for testing chemo‐photothermal therapies under NIR irradiation both in vitro (with increased intracellular uptake) and in vivo (with preferential tumor accumulation). Apoptosis‐dominant antitumor effects without significant necrosis were demonstrated by western blotting, and minimal systemic toxicity was confirmed by the weight‐change profiles of tumor‐bearing mice during treatment. Furthermore, NVs (lipid molecules instead of T) also were successfully fabricated using the FD reaction, and these NVs demonstrated NIR‐triggered drug release and efficient gene delivery. Thus, this work offers a generalizable on‐demand platform technology for fabricating numerous biofunctional NSs from combinations of photoionizable metals with bioactive molecules in a gaseous, single‐pass configuration.

## 4 Experimental Section

*Fabrication of TAuD NVs*: As shown in Figure S1 in the Supporting Information, Au AGs were produced via a laboratory‐made spark ablation reactor (volume, 42.8 cm^3^) comprising two Au rods (AU‐172561, Nilaco, Japan) and were continuously carried by nitrogen gas (99.999% purity; 3 L min^−1^) to a collision atomizer to be injected into a solution containing TD (0.10 mg of D and 26.75 mg of T in 1 mL ethanol) for the FD reaction. Hybrid droplets (e.g., Au AGs/TD) from the atomizer were exposed for 7.8 s to 185 nm wavelength photoirradiation (*E* = 6.2 eV, *I* = 0.14 J m^−2^ s^−1^; 3SC‐9‐A0, UVP, UK) to eject electrons from primary Au particles (work function, 5.1 eV) in the AGs. The surfaces of positively charged Au were electrostatically conjugated with negatively charged groups in TD, resulting in the reassembly of Au AGs forming TAuD NVs (Figure 1). The solvent was extracted from the hybrid droplets as they passed through a denuder containing pelletized activated carbons and silica gel. The NVs were charged with gaseous positive ions in a field charging configuration (pin (+4 kV)‐to‐ring (ground)), and subsequently collected on a polished aluminum rod under an electric field (−2.7 kV cm^−1^) via electrostatic attraction. The collecting rod was then immersed in PBS under ultrasonication (40 k Hz) for 5 min to release the NVs from the rod, forming an NV dispersion that was used in bioassays.

*Size Distribution in Aerosol and Aqueous States*: The size distribution of fabricated TAuD NVs, Au AG, and TD were determined using SMPS (3936, TSI, USA) to confirm quantitative interaction between the Au AG and TD. The flow rates of sampling and sheathing for SMPS measurements were 0.1 and 1.0 L min^−1^, respectively, and the scan time was 135 s. DLS (Nano‐ZS, Malvern Instruments, UK) was employed to measure the size distribution of TAuD NVs dispersed in PBS.

*Morphological Analysis*: The fabricated NVs were directly collected on a carbon‐coated copper grid (Tedpella, USA) via TEM grid filtration (Ineris, France) in a gaseous single‐pass configuration with no pretreatments. The grid was transferred to a holder for TEM analyses (CM‐100, FEI/Philips, USA) at increasing voltages in the range of 46–180 kV.

*Surface Chemistry and Light Absorption*: The surface chemistry of NVs was evaluated using FTIR (iS‐10, Thermo Electron, USA) in absorbance mode (1450–2300 cm^−1^) after NV deposition onto a polytetrafluoroethylene substrate (11807‐47‐N, Sartorius, Germany). Difference in the surface structure between the Au AG and TAuD were confirmed using XPS (Axis‐HIS, Kratos Analytical, Japan). TD incorporation onto Au surfaces was evaluated using Raman spectroscopy (T64000, HORIBA Jobin Yvon, Japan). The light absorption spectra of NVs dispersed in PBS and TD alone were measured using UV–vis spectroscopy (T60, PG Instruments, UK).

*Photothermal Activity*: The photothermal property of TAu NVs was activated using a fiber‐coupled infrared laser diode module (FC‐W‐808 nm‐30W, Changchun New Industries Optoelectronics Technology, China). Thermal images and temperature contours were recorded using a thermal camera (Therm‐App TH, Opgal Optronic Industries Ltd., Israel) fixed at 10 cm from the laser module. Three different concentrations of TAu NV in RPMI 1640 medium (100, 150, and 200 µg mL^−1^) were irradiated with a laser at constant power (power density, 2.5 W cm^−2^), and the resulting temperature profiles were recorded. The temperature profiles were also recorded as a function of irradiation intensity (1.8, 2.5, and 3 W cm^−2^) at a chosen NV concentration (150 µg mL^−1^).

*In Vitro Drug Release*: The in vitro D release from TAuD NVs was evaluated in ABS (pH 5.5; 0.14 m NaCl) and PBS (pH 7.4; 0.14 m NaCl). A TAuD dispersion sample (1 mL; 1 mg mL^−1^) (equivalent to 0.3 mg D) was placed in a dialysis bag (MWCO, 4000–6000 *D*_a_) (Spectra/Por, USA), successively dipped in a 50 mL tube containing 20 mL ABS (or PBS), and placed in a water bath shaker (HST‐205 SW, Hanbaek ST Co., Korea) at 100 strokes min^−1^ at 37 °C. Aliquots of the release medium (0.5 mL) were sampled at predetermined time intervals, followed by replenishment with an equal volume of fresh medium. The amount of D in the medium was determined using UV–vis spectrophotometry (U‐2800, PerkinElmer, USA). To investigate D release under laser irradiation, 1 mL TAuD was placed into a vial with a stirrer (400 RPM), diluted to 3 mL with PBS, and then irradiated from the top with a laser for 5 min. An aliquot (0.5 mL) was taken at predetermined time intervals and assayed for determining D concentration.

*Cytotoxicity*: Cytotoxicity measurements for free D, TAu, and TAuD samples were measured in MDA‐MB‐231 and MCF‐7 cells (i.e., human breast adenocarcinoma) via MTT assay in the presence or absence of NIR irradiation after 48 h incubation. Briefly, 1 × 10^4^ cells per well were plated into 96‐well microtiter plates (Becton Dickinson Labware, USA) and incubated for 12 h for cell attachment. After 48 h, the cells were washed and 100 µL MTT solution (1.25 mg mL^−1^) was added into each well. During a 4 h incubation in the dark, live cells produced violet‐colored formazan crystals as a product of MTT metabolism. The crystals were dissolved in 100 µL dimethyl sulfoxide, and the absorbance was measured at 570 nm using a microplate reader (Multiskan EX, Thermo Scientific, USA). Cell viability was calculated as *A*_sample_/*A*_control_ × 100%, where *A* is the absorbance at 570 nm.

*Intracellular Uptake*: Internalization of the prepared NVs by cells was observed using CLSM (TCS SP2, Leica Microsystems, Germany). MDA‐MB‐231 and MCF‐7 cells in 2 mL medium were seeded onto coverslips in 12‐well plates at a density of 5 × 10^4^ cells mL^−1^. Cells were incubated for 24 h to allow cell attachment, followed by the addition of 5 µg mL^−1^ coumarin‐6‐loaded TAu NV and 100 ng LysoTracker Red to each well. After 10 min incubation and subsequent medium removal, the coverslips were gently washed with PBS, fixed with 4% paraformaldehyde solution in the dark, mounted on glass slides, and sealed with glycerin. To confirm intracellular uptake, MDA‐MB‐231 or MCF‐7 cells (1 × 10^5^) in 2 mL medium were seeded onto 12‐well plates. After 12 h incubation, samples were incubated with TAuD for chosen periods of time. The cells were then washed with PBS, harvested by trypsinization, and resuspended in 1 mL PBS containing binding buffer for flow cytometric analyses using an FACS flow cytometer (BD FACS Verse, BD Biosciences, USA). Autofluorescence of untreated cells was used as an internal control.

*Apoptosis*: To compare the fractions containing preapoptotic, apoptotic, necrotic, and viable cells, 2 mL media containing MDA‐MB‐231 or MCF‐7 cells (2 × 10^5^) were placed in a 12‐well plate and incubated for 12 h. TAuD NVs and free D were added in the presence or absence of NIR irradiation. After 48 h, the cells were harvested by scraping, washed with PBS, and mixed with binding buffer. PE‐Annexin‐V and 7‐amino actinomycin D (2 µL each) were added, mixed gently, and left for 10 min in the dark. The treated cells were then diluted with binding buffer to a final volume of 1 mL, and apoptosis was analyzed using an FACS flow cytometer (BD FACS Verse, BD Bioscience, USA).

*Live/Dead Analysis*: MDA‐MB‐231 or MCF‐7 cells (3 × 10^5^ in 2 mL) were plated onto 12‐well dishes and incubated overnight for cell attachment. Following the addition of TAu (0.1 mg), cells were incubated for 3 h. After washing, the plate was placed 14 cm below the laser focus (spot size, 2 mm) and irradiated (4 W cm^−2^) for 3 min. After PBS removal, the cells were replenished with fresh medium and incubated for 3 h. Finally, the cells were stained with 2 × 10^−6^ m calcein‐AM (live cells, green fluorescence) and ethidium homodimer‐1 (dead cells, red fluorescence) in PBS and observed using an inverted fluorescence microscope (Eclipse Ti, Nikon, Japan).

*Cell Cycle Analysis*: The major cell cycle phases of MDA‐MB‐231 cells after treatment with free D and TAuD NVs were analyzed using the cell‐clock assay (Biocolor Ltd., UK). Cells were seeded onto 12‐well cell culture plates (2 × 10^5^ cells well^−1^) and incubated overnight to allow cell attachment. The cells were then treated with 0.1 µg mL^−1^ free D or TAuD (equivalent concentration) for 24 h. The cells were washed, replenished with fresh medium, and 150 µL cell‐clock dye reagent was added. After 1 h, the cells were washed, and images were obtained using an optical microscope (Eclipse Ti, Nikon, Japan). The cells in G2/M, S, and G0/G1 phases revealed color changes to dark blue, green, and yellow, respectively. The color pixels for each phase were quantified from photomicrographs using ImageJ software.

*Western Blot Analysis*: Western blot analyses were performed to investigate the expression of the apoptotic marker p53. MDA‐MB‐231 or MCF‐7 cells (4 × 10^5^ cells) were incubated for 12 h and treated with TAu for 24 h. The cells were then harvested, and the proteins were extracted from lysed cells and quantified using the bovine serum albumin protein assay kit. The proteins were separated on 10% polyacrylamide and electrophoretically transferred to a polyvinylidene fluoride membrane (Millipore, USA). After blocking the membranes with 5% skim milk in Tris‐buffered saline containing 1% Tween 20 (TBST), the membrane was incubated at 4 °C with primary antibodies against GAPDH and p53 (Santa Cruz Biotechnology, Inc., USA) for 12 h. The membranes were washed with TBST and incubated for 1 h with secondary antibodies. Proteins were detected using enhanced chemiluminescence agents on a luminescent image analyzer (LAS‐4000 mini, Fujifilm, Japan).

*In Vivo Antitumor Study*: MDA‐MB‐231 xenograft tumors were generated in 6‐week‐old female BALB/c nude mice by subcutaneous injection of 1 × 10^7^ MDA‐MB‐231 cells mixed with Corning Matrigel Matrix. When the tumors reached an approximate volume of 100 mm^3^, the mice were divided into five groups of 4–6 mice. Of these groups, four were subjected to either intravenous administration of free D (in saline), TAu (with NIR irradiation), or TAuD (with or without NIR irradiation). The samples were administered in a dose equivalent to 5 mg drug per kg body weight. The mice were exposed to NIR (3 W cm^−2^) for 3 min through the skin at 24 h after injection. The tumor dimensions were measured with digital calipers, and the tumor volume was calculated using the following formula: volume = 1/2 × (longest dimension) × (shortest dimension). All animal handling procedures were performed in accordance with the protocols approved by the Institutional Animal Ethical Committee, Yeungnam University, South Korea.

*Ex Vivo Distribution Measurement and In Vivo Photothermal Imaging*: The in vivo distribution of TAu in MDA‐MB‐231 xenografted BALB/c mice was investigated using a fluorescence in vivo imaging system (FOBITM, Neoscience, Korea). Cy5.5‐conjugated TAu was intravenously injected into xenografted mice. After 24 h, the mice were sacrificed by CO_2_ asphyxiation. The tumors and major organs were excised and scanned using FOBITM under the red channel. The fluorescent intensities were determined using NEOimage software.

Upon TAuD administration, in vivo photothermal imaging was performed using a digital thermal camera (Therm‐App TH, Israel). Briefly, mice were treated with either saline or TAuD. After 24 h, the tumors were exposed to NIR irradiation (4.0 W cm^−2^) for up to 5 min. Digital imaging was then performed to determine the photothermal effects of treatment.

*Gene Transfection*: HeLa cells were seeded at a density of 1 × 10^5^ cells well^−1^ in 24‐well plates. The cells were treated with the complex solution containing 2 mg pDNA (NV to pDNA, 10:1; w/w) for 4 h at 37 °C. Luciferase activity was measured with a luminometer (TD‐20/20, Promega, USA). The final luciferase activity was expressed as RLU mg^−1^ of protein. An inverted fluorescent microscope (DMI 4000 B, Leica Microsystems, Germany) was used to observe the green fluorescent protein expression in the cells.

## Conflict of Interest

The authors declare no conflict of interest.

## Supporting information

Supplementary
