# Supplementary material for: Photoinduced Rapid Transformation from Au Nanoagglomerates to Drug‐Conjugated Au Nanovesicles
Source: Adv Sci (Weinh). 2017 Dec 1;5(3):1700563. doi: 10.1002/advs.201700563 (PMC5867042; doi:10.1002/advs.201700563)
Supplement: Supplementary file 1 — Supplementary [file ADVS-5-1700563-s001.pdf]

Copyright WILEY-VCH Verlag GmbH & Co. KGaA, 69469 Weinheim, Germany, 2017.

ADVANCED SCIENCE
Open Access

Supporting Information

for *Adv. Sci.,* DOI: 10.1002/advs.201700563

Photoinduced Rapid Transformation from Au Nanoagglomerates to Drug-Conjugated Au Nanovesicles

Bijay Kumar Poudel, Jong Oh Kim, and Jeong Hoon Byeon**

Supporting Information

Photoinduced Rapid Transformation from Au Nanoagglomerates to Drug-Conjugated Au Nanovesicles

Bijay Kumar Poudel, Jong Oh Kim, and Jeong Hoon Byeon**

Dr. Bijay Kumar Poudel and Dr. Jeong Hoon Byeon

School of Mechanical Engineering, Yeungnam University, Gyeongsan 38541, Republic of Korea

Dr. Jong Oh Kim

College of Pharmacy, Yeungnam University, Gyeongsan 38541, Republic of Korea

*Corresponding authors: jongohkim@yu.ac.kr and postjb@yu.ac.kr

FIGURE S1

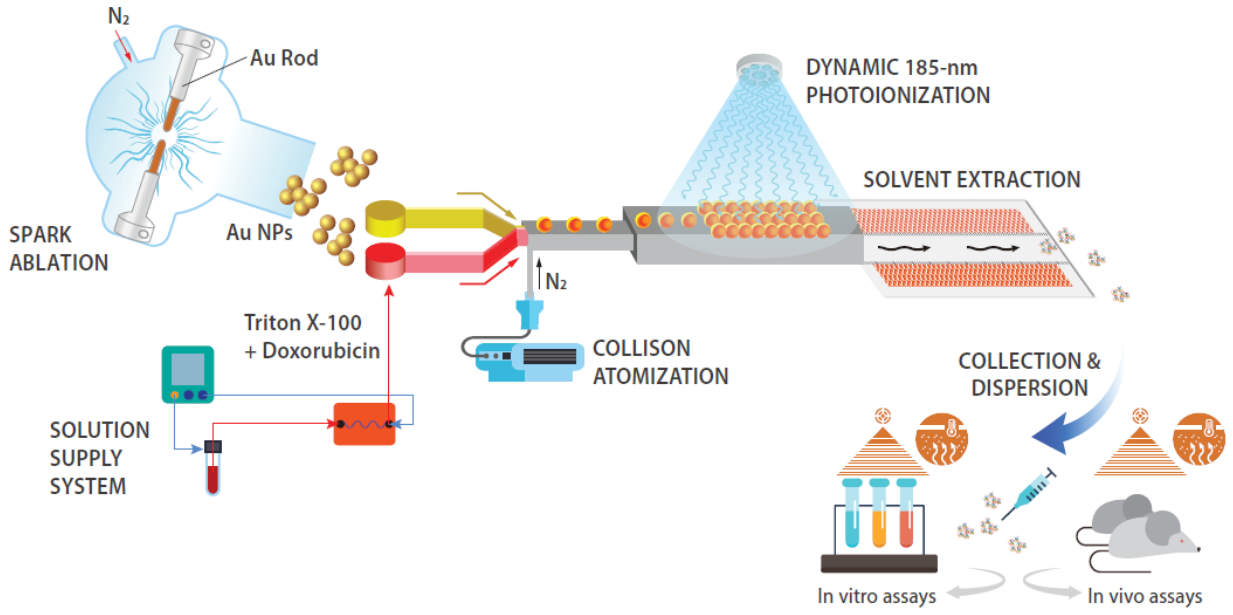

Reassembly of Au AGs to TAuD NVs through a gaseous, single-pass process under 185-nm UV irradiation. Au AGs were first prepared via spark ablation between two identical Au rods, and the AGs were then introduced into the TD solution before the orifice of a collison atomizer. The AGs and TD were sprayed out to form hybrid droplets, which were subsequently exposed to 185-nm UV (6.2 eV) for photoionization of Au AGs (5.1 eV) and subsequent electrostatic conjugation with TD in a denuder packed with pelletized activated carbons and silica gels, resulting in the formation of TAuD NVs. The NVs were electrostatically charged (+ field) and subsequently collected (– field) in an aerosol state and dispersed in PBS for *in vitro* and *in vivo* bioassays.

FIGURE S2

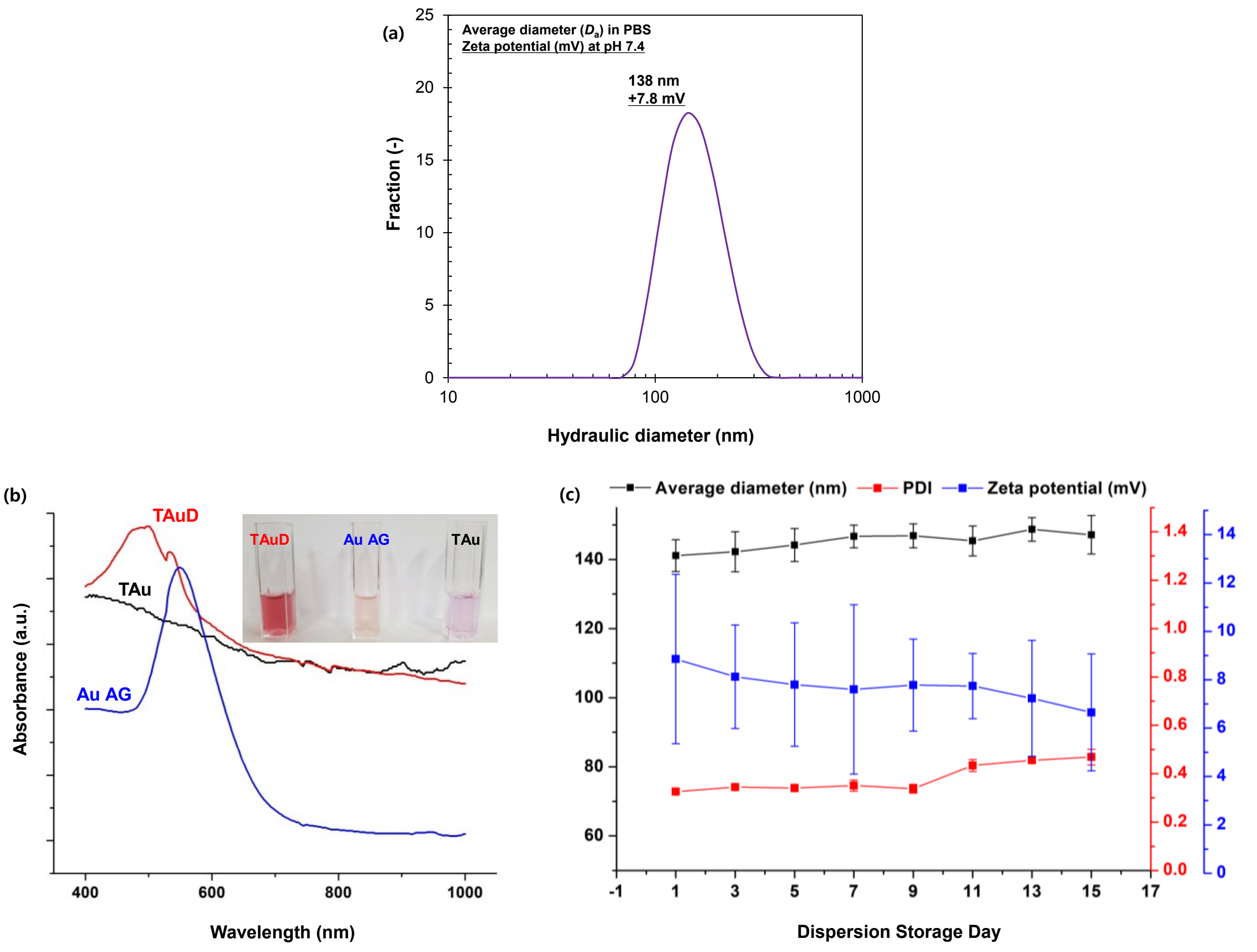

(a) DLS particle size distribution, zeta potential, and (b) UV-vis spectra (including TD) of TAuD NVs dispersed in PBS (0.2 mg mL^{-1}). The absorbances of TAuD, TAu, and Au AG samples at 808 nm were 0.254, 0.266, and 0.11, respectively, and the analogous data at 632 nm were 0.307, 0.295, and 0.166, respectively. Inset in (b) shows TAuD, Au AGs, and TAu dispersions in PBS. (c) Changes in average diameter, polydispersity index (PDI), and zeta potential for dispersion storage (15 days) of TAuD NVs. For 15-day measurements after they were dispersed in PBS, there were no significant changes in the size distribution and zeta potential.

FIGURE S3

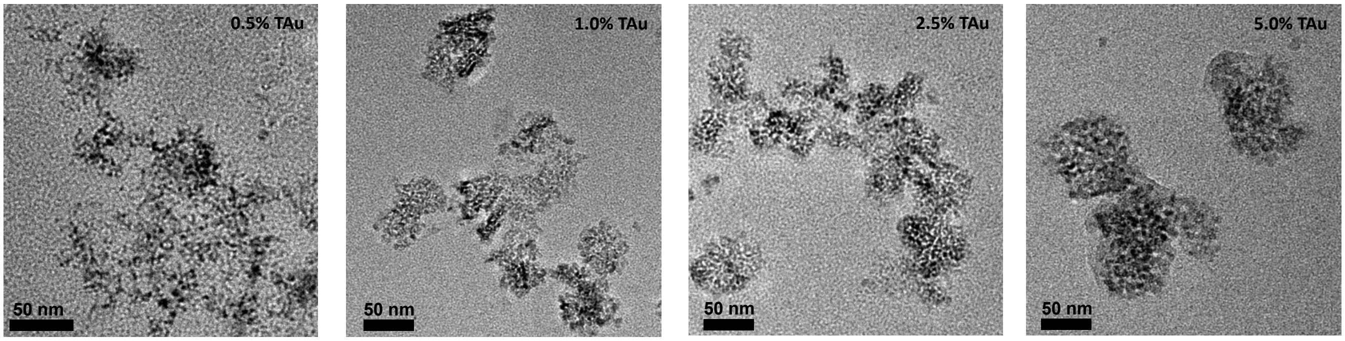

TEM images of reassembled Au AGs incorporating different T solutions: 5.35 (0.5 v·T/v·ethanol%), 10.70 (1.0 v/v%), 26.75 (2.5 v/v%), and 53.50 (5.0 v/v%) mg·T mL^{-1}·ethanol. Increasing T concentrations resulted in vesicles assuming a more tightened configuration.

FIGURE S4

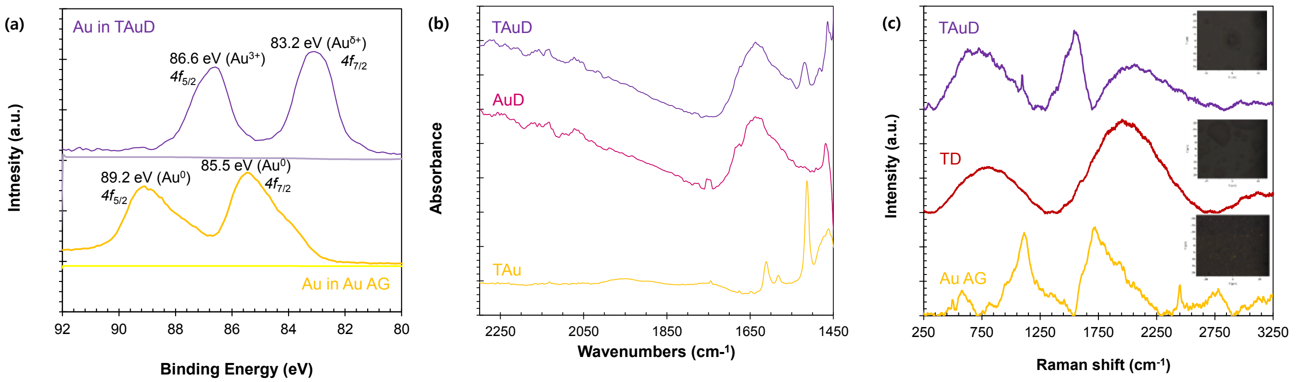

(a) XPS Au spectra in TAuD and Au AG, (b) FTIR spectra of TAuD, AuD, and TAu, and (c) Raman spectra of TAuD, TD, and Au AG (insets show optical microscope specimens on glass discs for measurements).

FIGURE S5

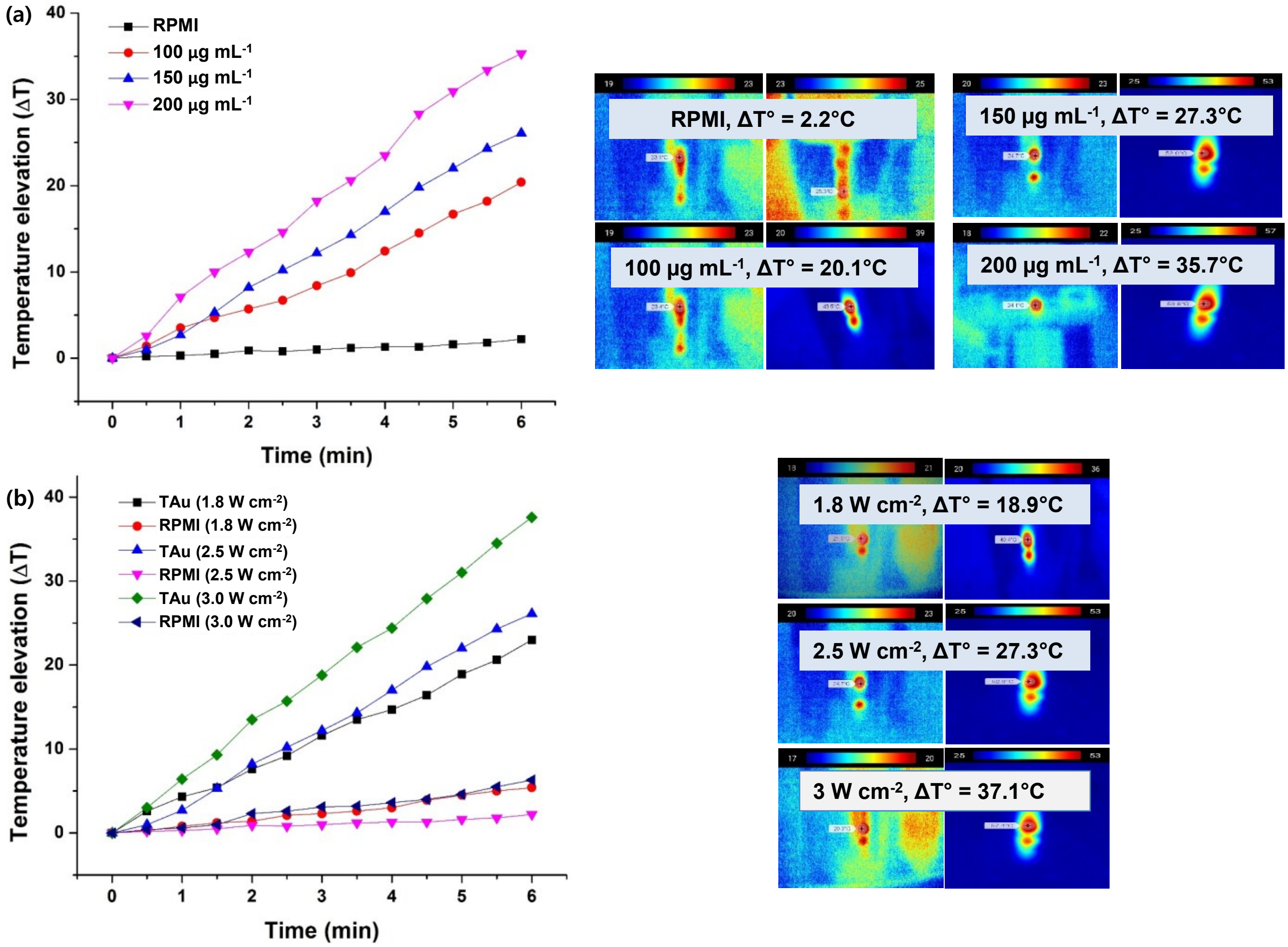

(a) Concentration-dependent (at 2.5 W cm^{-2}) and (b) 808-nm NIR irradiation intensity-dependent (at 150 µg mL^{-1}) temperature elevation profiles of TAu and RPMI (control) for 6 min. Temperature contours for each condition are included. Temperatures near the laser beam spot significantly increased in response to irradiation.

FIGURE S6

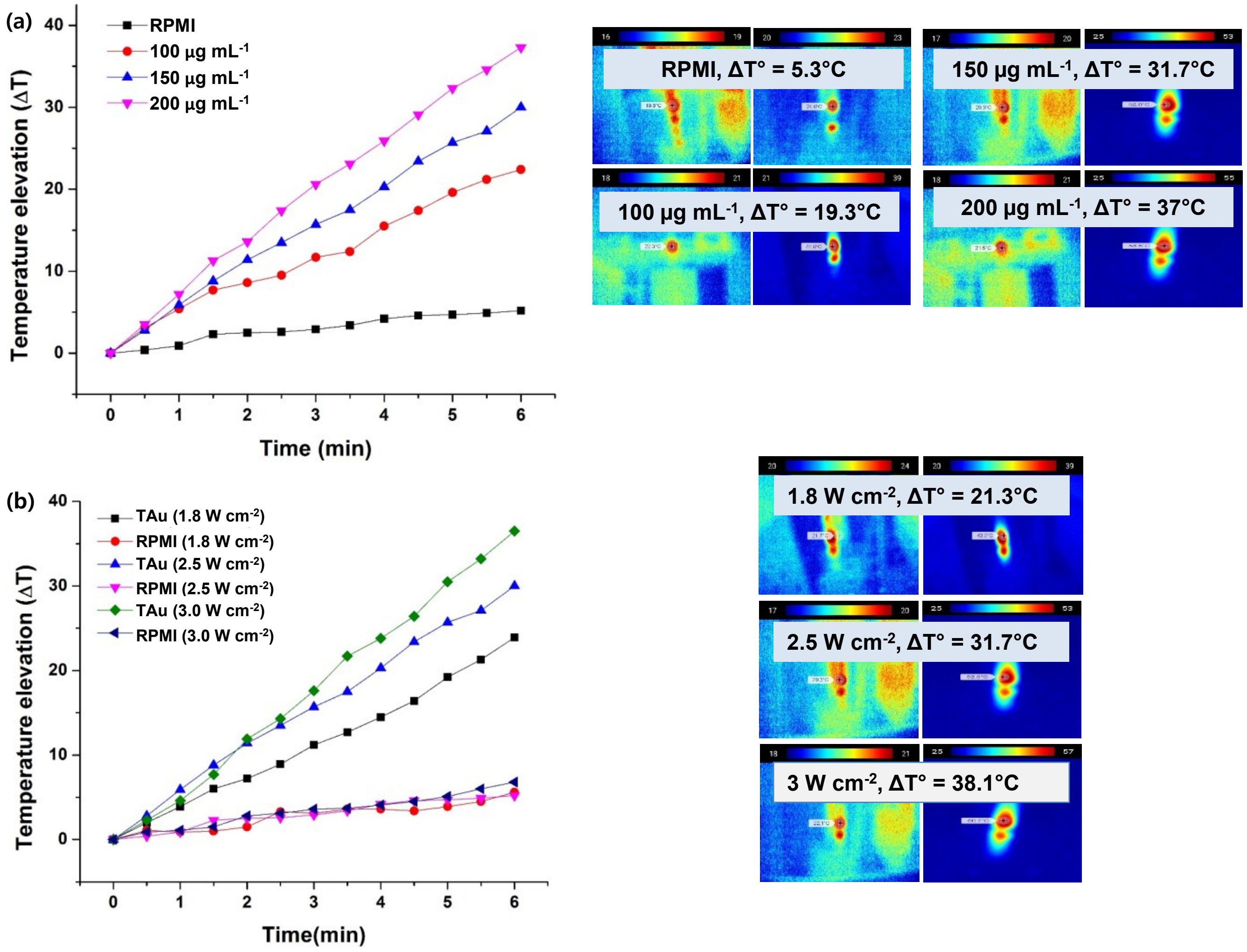

(a) Concentration-dependent (at 2.5 W cm^{-2}) and (b) 632-nm NIR irradiation intensity-dependent (at 150 μg mL^{-1}) temperature elevation profiles of TAu and RPMI (control) for 6 min. Temperature contours for each condition are included. Temperatures near the laser beam spot increased significantly in response to irradiation.

FIGURE S7

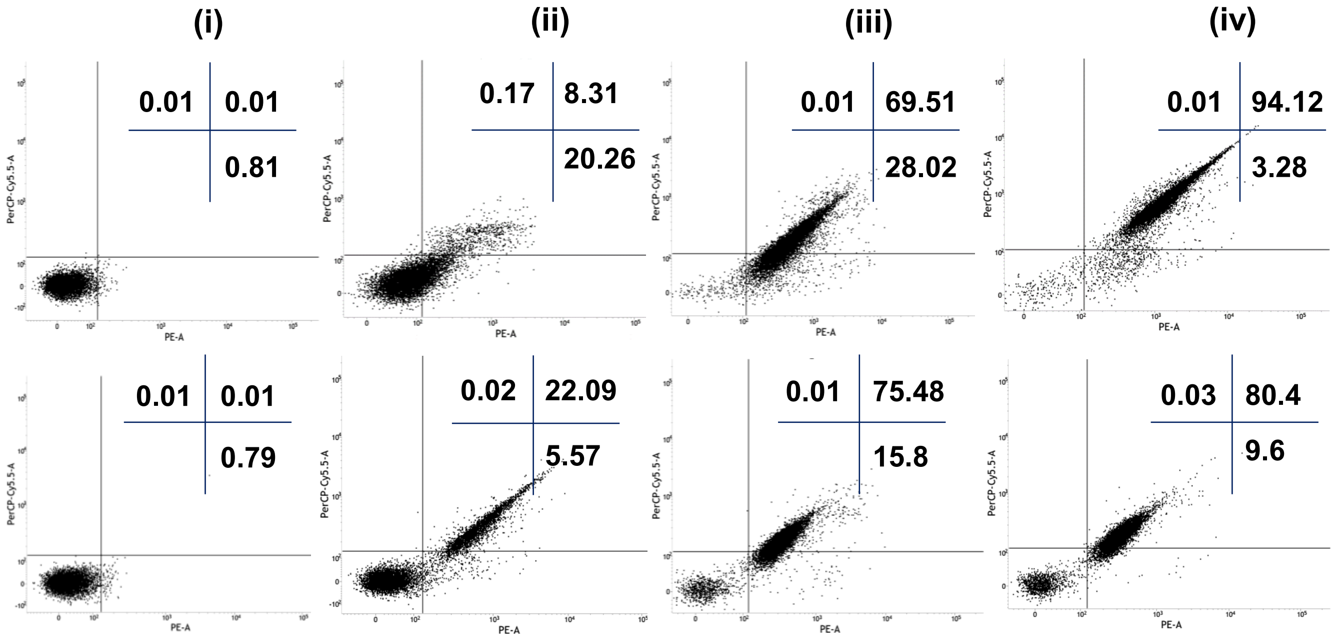

FACS results to determine cellular apoptosis of MCF-7 (top) and MDA-MB-231 (bottom) cells following treatment with (i) control, (ii) free D, (iii) TAu + NIR, and (iv) TAuD + NIR.

FIGURE S8

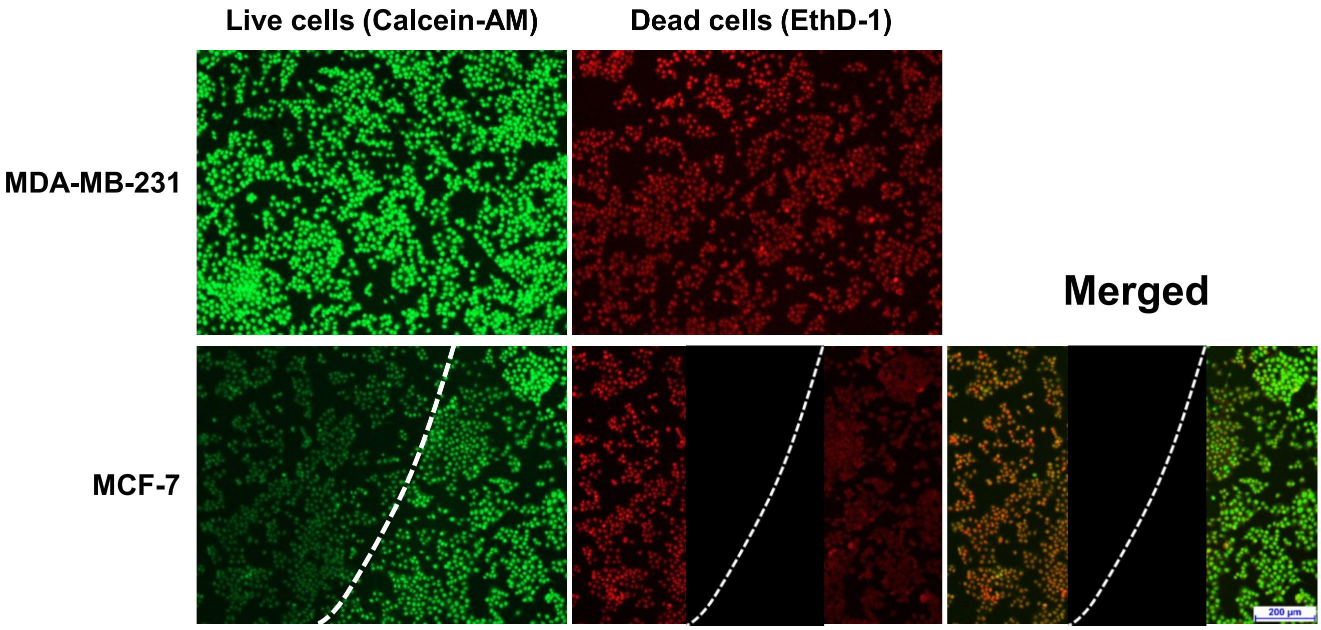

Inverted fluorescence microscope images from live/dead assays. The displayed images show live cells (green, stained with calcein-AM) and dead cells (red, stained with EthD-1) after NIR irradiation (4 W cm^{-2}) for 5 min. Dashed lines demarcate the areas of irradiation.

FIGURE S9

(a)
(b)
(c)

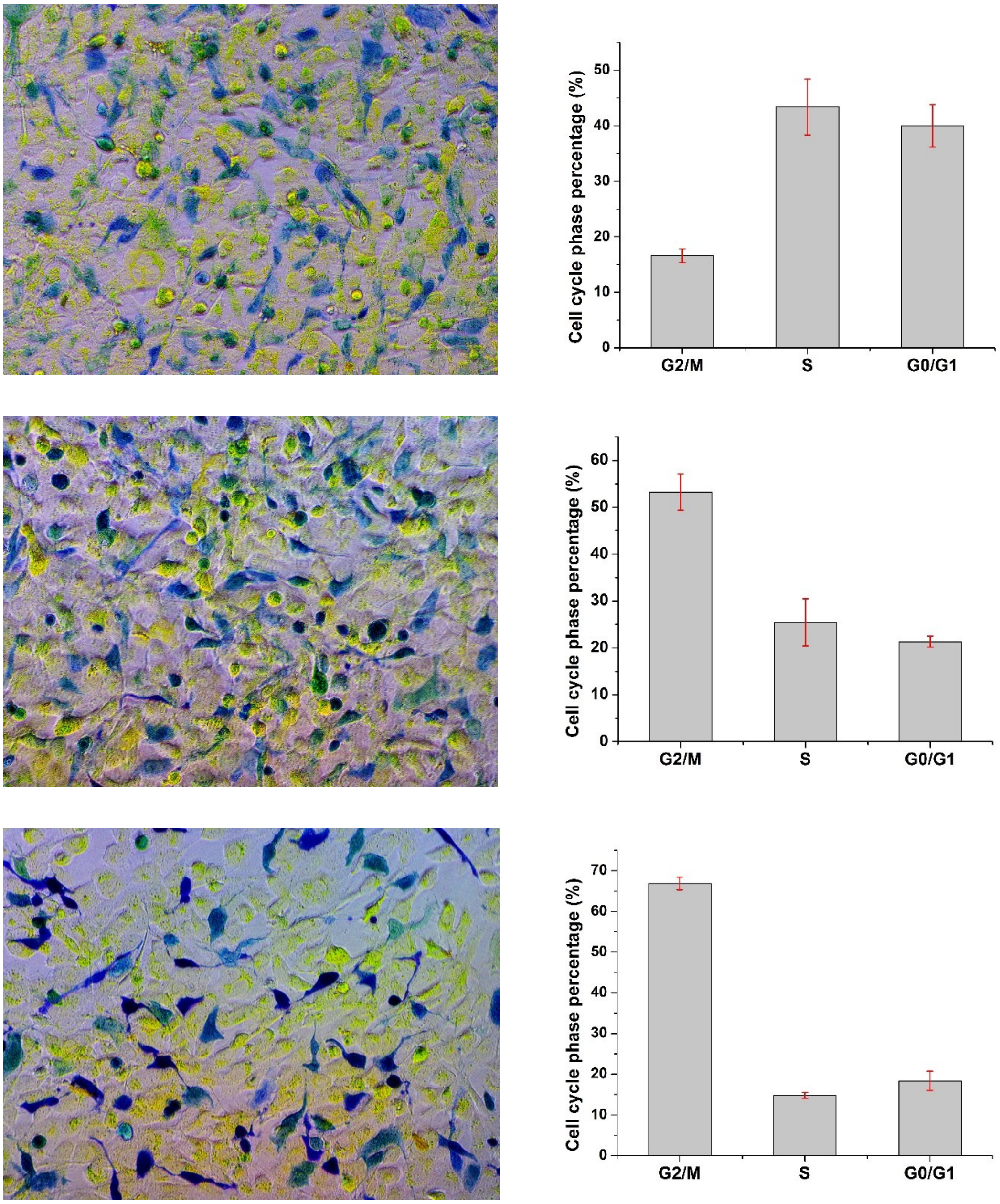

Optical microscope images from cell cycle analyses. Cell-clock labeling in MDA-MB-231 cells was performed after 24-h incubation with (a) control, (b) free D, and (c) TAuD.

FIGURE S10

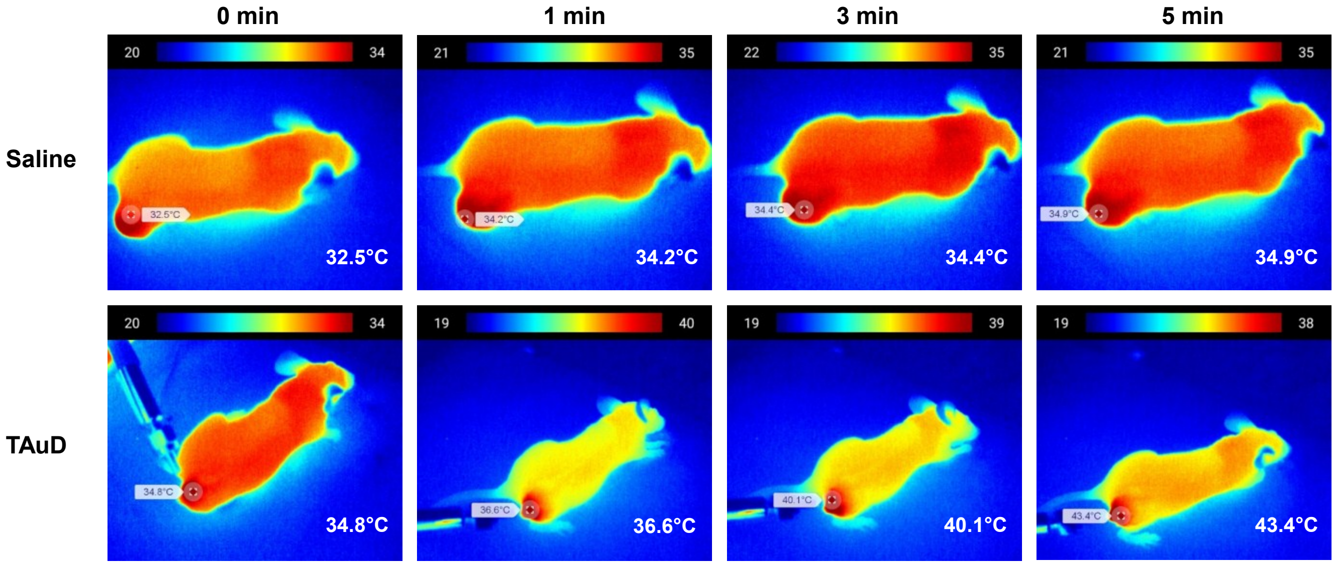

In vivo photothermal imaging during NIR irradiation of tumors in mice pretreated with saline and TAuD.
